# Accuracy of Diagnosing Heparin-Induced Thrombocytopenia

**DOI:** 10.1001/jamanetworkopen.2024.3786

**Published:** 2024-03-26

**Authors:** Emil List Larsen, Henning Nilius, Jan-Dirk Studt, Dimitrios A. Tsakiris, Andreas Greinacher, Adriana Mendez, Adrian Schmidt, Walter A. Wuillemin, Bernhard Gerber, Prakash Vishnu, Lukas Graf, Johanna A. Kremer Hovinga, Jens P. Goetze, Tamam Bakchoul, Michael Nagler

**Affiliations:** 1 Department of Clinical Biochemistry, Copenhagen University Hospital–Rigshospitalet, Copenhagen, Denmark; 2 Department of Clinical Chemistry, Inselspital, Bern University Hospital, Bern, Switzerland; 3 Graduate School for Health Sciences, University of Bern, Bern, Switzerland; 4 Division of Medical Oncology and Hematology, University Hospital Zurich, Zurich, Switzerland; 5 Diagnostic Haematology, Basel University Hospital, Basel, Switzerland; 6 Department of Transfusion Medicine, Institute of Transfusion Medicine, University Medicine Greifswald, Greifswald, Germany; 7 Department of Laboratory Medicine, Kantonsspital Aarau, Aarau, Switzerland; 8 Institute of Laboratory Medicine and Clinic of Medical Oncology and Hematology, Municipal Hospital Zurich Triemli, Zurich, Switzerland; 9 Division of Hematology and Central Hematology Laboratory, Cantonal Hospital of Lucerne and University of Bern, Switzerland; 10 Clinic of Hematology, Oncology Institute of Southern Switzerland, Bellinzona, Switzerland; 11 Fred Hutchinson Cancer Center, University of Washington, Seattle; 12 Cantonal Hospital of St Gallen, St Gallen, Switzerland; 13 Department of Hematology and Central Hematology Laboratory, Inselspital, Bern University Hospital, Bern, Switzerland; 14 Department of Biomedical Sciences, Faculty of Health, Copenhagen University, Copenhagen, Denmark; 15 Centre for Clinical Transfusion Medicine, University Hospital of Tübingen, Tübingen, Germany; 16 University of Bern, Bern, Switzerland

## Abstract

**Importance:**

Heparin-induced thrombocytopenia (HIT) is a life-threatening condition that requires urgent diagnostic clarification. However, knowledge of the diagnostic utility of the recommended diagnostic tests is limited in clinical practice.

**Objective:**

To evaluate the current diagnostic practice for managing the suspicion of HIT.

**Design, Setting, and Participants:**

This prospective diagnostic study was conducted from January 2018 to May 2021 among consecutive patients with suspected HIT from 11 study centers in Switzerland, Germany, and the United States. Detailed clinical data and laboratory information were recorded. Platelet factor 4/heparin antibodies were quantified using an automated chemiluminescent immunoassay (CLIA). A washed-platelet heparin-induced platelet activation (HIPA) test was used as a reference standard to define HIT.

**Exposures:**

Suspicion of HIT.

**Main Outcomes and Measures:**

The primary outcome was the diagnostic accuracy of the 4Ts score, the CLIA, and the recommended algorithm serially combining both tests.

**Results:**

Of 1448 patients included between 2018 and 2021, 1318 were available for the current analysis (median [IQR] age, 67 [57-75] years; 849 [64.6%] male). HIPA was positive in 111 patients (prevalence, 8.4%). The most frequent setting was intensive care unit (487 [37.0%]) or cardiovascular surgery (434 [33.0%]). The 4Ts score was low risk in 625 patients (46.8%). By 2 × 2 table, the numbers of patients with false-negative results were 10 (9.0%; 4Ts score), 5 (4.5%; CLIA), and 15 (13.5%; recommended diagnostic algorithm). The numbers of patients with false-positive results were 592 (49.0%; 4Ts score), 73 (6.0%; CLIA), and 50 (4.1%; recommended diagnostic algorithm), respectively.

**Conclusions and Relevance:**

In this diagnostic study of patients suspected of having HIT, when the recommended diagnostic algorithm was used in clinical practice, antibody testing was required in half the patients. A substantial number of patients were, however, still misclassified, which could lead to delayed diagnosis or overtreatment. Development of improved diagnostic algorithms for HIT diagnosis should be pursued.

## Key Points

**Question:**

Is the current diagnostic practice for suspected heparin-induced thrombocytopenia (HIT) accurate?

**Findings:**

In this diagnostic study of 1318 patients suspected of having HIT, the 4Ts score produced 9.0% false negatives; chemiluminescent immunoassay produced 4.5%; and the recommended algorithm (4Ts score followed by chemiluminescent immunoassay) produced 13.5%. These same tests produced 49.0%, 6.0%, and 4.1% false positives, respectively.

**Meaning:**

In this study, a substantial number of patients with suspected HIT were misclassified, which could lead to delayed diagnosis or overtreatment.

## Teaser

This diagnostic study evaluates the current diagnostic practices for managing suspected heparin-induced thrombocytopenia.

## Introduction

The diagnostic utility of recommended diagnostic tests in daily practice often does not match published study results, and little is known about tests used to diagnose heparin-induced thrombocytopenia (HIT). Immune-mediated HIT is a life-threatening complication seen after heparin administration.^1,2,3,4^ Immunogenic complexes are generated from IgG antibodies targeting platelet factor 4 (PF4)/heparin-complexes.^5,6,7^ In turn, these complexes activate platelets through FcγRIIA receptors and cause platelet aggregation.^8^ As phenotype, a triad of signs and symptoms are common: (1) thrombocytopenia, (2) severe thrombosis, and (3) a typical timing of platelet count reduction following heparin administration.^8,9^ HIT is uncommon and occurs in diverse settings, making it unfamiliar to the clinician involved.^10^ It is, however, imperative to know when to suspect HIT and how to handle it correctly to prevent severe thromboembolic complications.^8^

The workup of suspected HIT is challenging because the available diagnostic tools are associated with major drawbacks. The 4Ts score is a clinical scoring system aiming to determine the clinical (pretest) probability of HIT (Table 1).^11,12^ Despite a meta-analysis^13^ stating that the 4Ts score has a high negative predictive value, the sensitivity of the score in clinical practice has been challenged by some studies.^10,14^ Furthermore, the positive predictive value was low, suggesting HIT in a large proportion of patients. Several enzyme-linked immunosorbent assays and rapid immunoassays have been developed to better identify and quantify PF4/heparin antibodies.^15^ In general, most assays have a high sensitivity and negative predictive value.^15,16^ Of note, not all PF4/heparin antibodies activate platelets and cause HIT, thus limiting the specificity and positive predictive value of immunoassays.^8^ Functional tests, eg, the washed-platelet heparin-induced platelet activation assay (HIPA) or serotonin release assay (SRA), can verify platelet-activating antibodies, thus confirming the presence of HIT.^10,17^ These functional tests are, however, technically challenging, and can only be performed in a few specialized laboratories. Thus, scientific societies recommend using the 4Ts score first. In case of intermediate or high risk, PF4/heparin antibodies should be determined using a validated assay.^15^ If PF4/heparin antibodies are also present, a functional test should then be performed to consolidate the diagnosis.^11,12^ However, little is known about the current practices and the performance of the recommended diagnostic algorithm in clinical practice. As part of a prospective, multicenter cohort study, we aimed to assess the diagnostic accuracy of currently recommended tests for HIT in clinical practice: (1) the 4Ts score, (2) the AcuStar HIT-IgG chemiluminescent immunoassay (CLIA), and (3) the diagnostic algorithm serially combining 4Ts score and CLIA.

**Table 1.** 4Ts Score^a^

Category	2 Points	1 Point	0 Points
Thrombocytopenia	Platelet count falls >50% and platelet nadir ≥20 × 10^3^/μL	Platelet count falls 30%-50% or platelet nadir 10-19 × 10^3^/μL	Platelet count falls <30% or platelet nadir <10 × 10^3^/μL
Timing of platelet count fall following heparin administration	5-10 d (Clear onset) or ≤1 d, if prior heparin administration (within 30 d)	5-10 d (Unclear onset, eg, missing platelet count), >10 d, or ≤1 d, if prior heparin administration (within 30-100 d)	≤4 d, With no recent heparin administration (within 100 d)
Thrombosis or another sequela	New thrombosis, skin necrosis, or acute systemic reaction after intravenous unfractionated heparin bolus	Progressive or recurrent thrombosis, nonnecrotizing, or suspected thrombosis	None
Other causes of thrombocytopenia	None	Possible	Definite

SI conversion factor: To convert platelets to cells × 10^9^ per liter, multiply by 1.

^a^ A score of 0 to 3 points indicates low risk of heparin-induced thrombocytopenia; 4 to 5 points, intermediate risk; 6 to 8 points, high risk. Compiled from Lo et al.^22^

## Methods

### Study Design, Setting, and Participants

This analysis is part of Towards Precise and Rapid Diagnosis of Heparin-Induced Thrombocytopenia: A Prospective, Multicentre Cohort Study (TORADI-HIT), which includes consecutive patients with suspected HIT from 11 tertiary hospitals localized in Switzerland, Germany, or the United States. A detailed description of study design, setting, participants, study procedures, and collection of data are provided in previous publications that have addressed (1) the development of a decision support tool^18^ and (2) the consistency of different heparin/PF4 immunoassays.^19^ Inclusion criteria were (1) suspected HIT (PF4/heparin antibodies ordered, 4Ts score applied, or consultancy service requested), (2) aged 18 years or older, and (3) written informed consent provided. Patients were included between January 2018 and May 2021. Patients were excluded in case of insufficient sample material, insufficient clinical data, or refused consent. The study was approved by all ethical committees (ie, Kantonale Ethikkommission Bern) and conducted in accordance with the Declaration of Helsinki. The manuscript was prepared following the Standards for Reporting of Diagnostic Accuracy (STARD) guideline.

### Data Collection

A set of prespecified clinical characteristics and laboratory test results at diagnosis were collected by specially trained study nurses into an electronic case report form (REDCap database).^18,20,21^ In particular, the individual items of the 4Ts score were recorded as they were available to the treating physicians at the time of diagnosis; 4Ts scores were scored by the attending physician in conjunction with the consultant service. Specially trained study nurses transferred this data to the electronic case report form. If gross errors were found, they were corrected in consultation with the attending physicians and the principal investigator. In any case, only data available to the treating physicians at the time of diagnosis was used. The 4Ts score is an established scoring system to determine the clinical (pretest) probability of HIT (Table 1): 0 to 3 points indicates low risk; 4 to 5 points, intermediate risk; and 6 to 8 points, high risk.^22^

A residual serum sample was obtained at the time of diagnosis, and it was frozen at −80 °C and transported on dry ice to the central laboratory Department of Clinical Chemistry, Inseptal, Bern University Hospital, Bern Switzerland. Follow-up was continued until discharge.

### Determination of the CLIA

Within 1 week after arrival of the sample, PF4/heparin antibodies were quantified using a CLIA (HemosIL AcuStar HIT-IgG; Instrumentation Laboratory). CLIA was conducted on a BIO-FLASH (Inova Diagnostics) analyzer according to the manufacturers’ instructions, as previously described.^23^ In brief, the assay was calibrated using calibrator 1 and calibrator 2 from the manufacturer, and samples were thawed rapidly at 37 °C. Internal quality controls were used before each run. The cutoff defined by the manufacturer was used, and samples with a result of 1.00 U/mL or higher were considered positive.

### HIPA Test

As a reference standard, the presence of HIT was determined by a washed-platelet functional assay, the HIPA.^11,18^ Like the SRA, the washed-platelet HIPA is recognized as the criterion standard of reference by major scientific societies and many authors.^10,16,17,18,19,22,23^ The HIPA was conducted as described in detail previously.^17^ In brief, serum was mixed with washed platelets from 4 different donors and placed on a microplate. Buffer, 0.2 IU/mL of low-molecular-weight heparin, or 100 IU/mL unfractionated heparin was added to the sample. The microplate was incubated for 45 minutes on a magnetic stirrer plate with 2 steel balls per well at 600 rpm. Platelet activation was observed every 5 minutes. The test was considered positive if aggregation occurred within 30 minutes in the presence of 0.2 IU/mL low-molecular-heparin, but not in the presence of 100 IU/mL heparin in at least 2 donors.^17^

### Statistical Analysis

Patient characteristics were reported grouped by HIPA test results as median with IQR because Gaussian criteria were not met. A 2 × 2 table was created, and diagnostic accuracy measures of (1) the 4Ts score, (2) the CLIA, and (3) the recommended diagnostic algorithm (4Ts score followed by CLIA) were calculated based on predefined cutoff values.^11,24^ Sample size considerations were reported in detail previously.^18^ R version 4.1.0 (R Project for Statistical Computing) was used for statistical analysis and graphical illustrations of data. Sensitivity, specificity, positive predictive, and negative predictive values with 95% CIs were calculated using the epiR package. Area under the curve (AUC) on receiver operating characteristic (ROC) curves was calculated using the pROC package.^25^

## Results

### Patient Characteristics

Between 2018 and 2021, a total of 1448 patients were included from 11 study centers (eFigure in Supplement 1). After exclusion of 130 patients because of insufficient clinical information or insufficient serum samples, 1318 patients were eligible for the present analysis (median [IQR] age, 67 [57-75] years; 849 [64.6%] male). HIPA was positive in 111 patients, corresponding to a prevalence of 8.4%. The most common settings were intensive care unit (487 [37.0%]) and cardiovascular surgery (434 [33.0%]) (Table 2). The 4Ts score was categorized as low risk in 625 patients (46.8%), intermediate risk in 611 patients (46.9%), and high risk in 82 patients (6.2%) (Table 2). Unfractionated heparin was used in 1055 patients (88.3%), and new, recurrent, or progressive thromboembolism was present in 357 patients (27.1%).

**Table 2.** Patient Characteristics^a^

Characteristic	Patients, No. (%)			Missing values, %
	All patients (n = 1318)	HIT		
		Positive (n = 111)	Negative (n = 1207)	
Sex				
Female	466 (35.4)	45 (40.5)	421 (34.9)	0.2
Male	849 (64.6)	66 (59.5)	783 (65.0)	
Age, median (IQR), y	67 (57-75)	65 (56-75)	67 (58-75)	0.1
Setting				
Intensive care unit	487 (37.0)	38 (34.2)	449 (37.2)	0.1
Internal medicine	255 (19.4)	14 (12.6)	241 (20.0)	
Major trauma	10 (0.8)	6 (5.4)	4 (0.3)	
Postoperative: cardiovascular surgery	434 (33.0)	46 (41.4)	388 (32.2)	
Postoperative: other surgery	119 (9.0)	6 (5.4)	113 (9.4)	
Other	12 (0.9)	1 (0.9)	11 (0.9)	
4Ts score				
Low risk (0-3)	625 (47.4)	10 (9.0)	615 (51.0)	0.0
Intermediate risk (4-5)	611 (46.4)	65 (58.6)	546 (45.2)	
High risk (6-8)	82 (6.2)	36 (32.4)	46 (3.8)	
Thrombosis^b^				
Deep vein thrombosis	31 (2.4)	5 (4.5)	26 (2.2)	0.0
Pulmonary embolism	68 (5.2)	10 (9.0)	58 (4.8)	
Other venous thrombosis	90 (6.8)	14 (12.6)	76 (6.3)	
Stroke	38 (2.9)	6 (5.4)	32 (2.7)	
Myocardial infarct	12 (0.9)	3 (2.7)	9 (0.7)	
Skin necrosis	9 (0.7)	1 (0.9)	8 (0.7)	
Other arterial thrombosis	109 (8.3)	13 (11.7)	96 (8.0)	
No	961 (72.9)	59 (53.2)	902 (74.7)	
Heparin administration (last 2 weeks)				
Unfractionated heparin	1055 (80.0)	98 (88.3)	957 (79.3)	0.0
Low-molecular-weight heparin	567 (43.0)	45 (40.5)	522 (43.2)	

Abbreviation: HIT, heparin-induced thrombocytopenia.

^a^ Patient characteristics have previously been published.^18,19^

^b^ New, recurrent, or progressive thromboembolism.

### Diagnostic Accuracy

**Figure 1.**
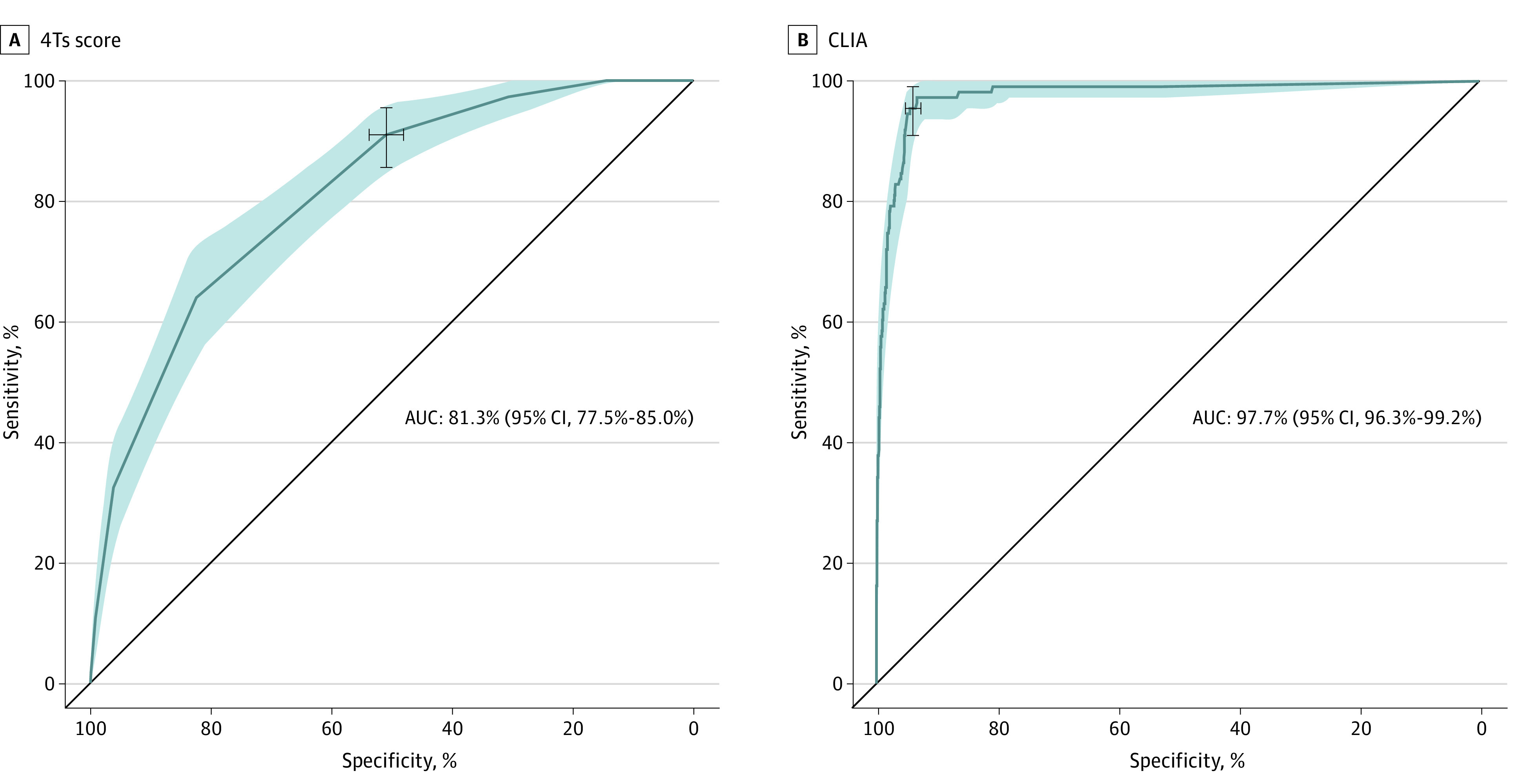
Receiver Operating Characteristic Curves Receiver operating characteristic curve of 4Ts score (A) and chemiluminescent immunoassay (CLIA; B) as with 95% CIs (shaded area). Diagnostic thresholds with 95% CIs are marked. AUC indicates area under the curve.

**Figure 2.**
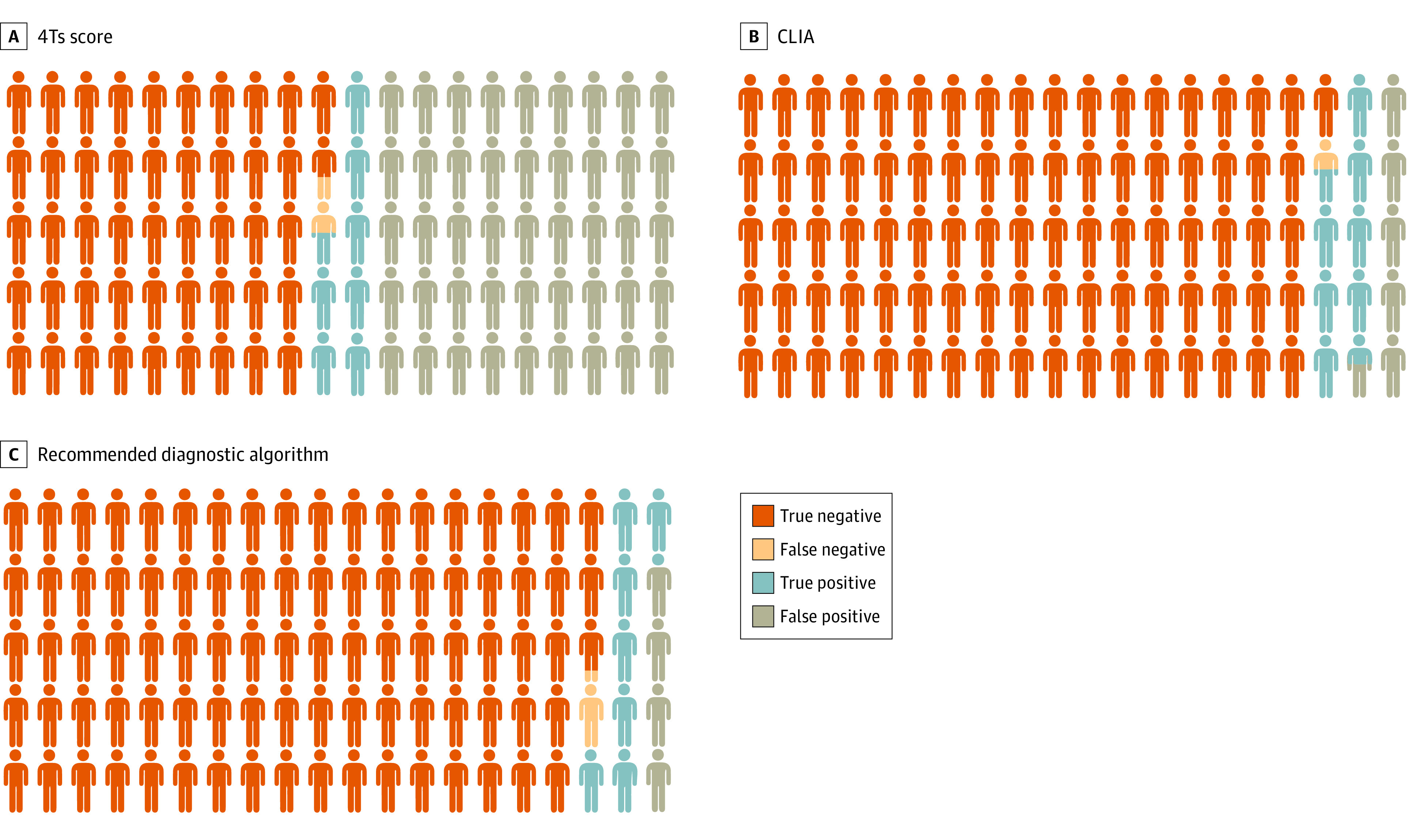
Diagnostic Performance of Diagnostic Tests for Heparin-Induced Thrombocytopenia in Clinical Practice Proportion of true-negative (dark orange), false-negative (light orange), true-positive (blue), and false-positive (gray) results are given using the 4Ts score (A), chemiluminescent immunoassay (CLIA; B), and the recommended diagnostic algorithm (C). Created with Biorender.com.

The 4Ts score correctly classified 101 patients as HIT positive and 615 as HIT negative (Table 3). The numbers of false negatives and false positives were 10 (9.0%) and 592 (49.0%), respectively. Baseline characteristics of the individuals with a false-negative 4Ts score are available in the eTable in Supplement 1. The CLIA correctly identified 106 patients as HIT positive and 1134 as HIT negative. The numbers of false negatives and false positives were 5 (4.5%) and 73 (6.0%), respectively. The currently recommended diagnostic algorithm (4Ts score followed by CLIA in case of an intermediate- or high-risk 4Ts score) correctly identified 96 patients as HIT positive and 1157 as HIT negative. The numbers of false negatives and false positives were 15 (13.5%) and 50 (4.1%), respectively. Sensitivities and specificities are shown in Table 3. Of note, the recommended diagnostic algorithm missed 13.5% of patients with HIT. In our dataset, the positive and negative predictive values were 14.6% (5% CI, 12.0%-17.4%) and 98.4% (95% CI, 97.1%-99.2%) for the 4Ts score, 59.2% (95% CI, 51.6%-66.5%) and 99.6% (95% CI, 99.0%-99.9%) for the CLIA, and 65.7% (95% CI, 57.5%-73.4%) and 98.7% (95% CI, 97.9%-99.3%) for the recommended diagnostic algorithm. ROC curves of all diagnostic tests are shown in Figure 1. The area under the ROC curve was 81.3% (95% CI, 77.5%-85.0%) for the 4Ts score and 97.7% (95% C,: 96.3%-99.2%) for the CLIA. Figure 2 illustrates the proportion of false negatives, false positives, true negatives, and true positives as observed in our representative population.

**Table 3.** Diagnostic Accuracy of the 4Ts score, CLIA, and the Recommended Diagnostic Algorithm Serially Combining 4Ts Score and CLIA

Test	Individuals, No.	No. (%)^a^				% (95% CI)			
		Negatives		Positives		Sensitivity	Specificity	PPV	NPV
		True	False	True	False				
4Ts score	1318	615 (51.0)	10 (9.0)	101 (91.0)	592 (49.0)	91.0 (84.1-95.6)	51.0 (48.1-53.8)	14.6 (12.0-17.4)	98.4 (97.1-99.2)
CLIA	1318	1134 (94.0)	5 (4.5)	106 (95.5)	73 (6.0)	95.5 (89.8-98.5)	94.0 (92.5-95.2)	59.2 (51.6-66.5)	99.6 (99.0-99.9)
Recommended diagnostic algorithm	1318	1157 (95.9)	15 (13.5)	96 (86.5)	50 (4.1)	86.5 (78.7-92.2)	95.9 (94.6-96.9)	65.8 (57.5-73.4)	98.7 (97.9-99.3)

Abbreviations: CLIA, chemiluminescent immunoassay; NPV, negative predictive value; PPV, positive predictive value.

^a^ Percentages are given according to the 2 × 2 table.

## Discussion

In this study, we examined the current diagnostic practice of managing clinical suspicion of HIT in a clinical setting using a prospective multicenter approach. By applying the current diagnostic algorithm, nearly half the patients would not require antibody testing. However, all tests, and especially the current diagnostic algorithm, still misclassify a concerning number of patients as either false negative or false positive.

Some studies have addressed the utility of current diagnostic instruments for HIT in clinical practice, and our results are essentially in line with these preliminary investigations. Linkins and colleagues^14^ prospectively studied 526 of 1781 patients with a requested PF4/heparin assay using the SRA as reference standard (the prevalence of HIT was 6.1%). The sensitivity of the 4Ts score, representing the first step in the currently recommended algorithm, was only 81.3%, which would have missed a remarkable proportion of HIT patients.^14^ Similar to our results, the sensitivity of the PF4/heparin immunoassay was higher (100%; using rapid particle gel immunoassay).^14^ These results are, however, in striking contrast to a number of studies that report much higher sensitivity of the 4Ts score, which might potentially not reflect what happens in clinical practice.^13^ Similar to other studies, we have analyzed the accuracy of PF4/heparin immunoassays and found high sensitivities.^15,24,26,27^ In another study conducted in clinical practice, Gallo and colleagues^28^ retrospectively included 319 patients in a 30-hospital US health care system, in which a previously implemented clinical decision support (CDS) fired during HIT immunoassay order entry indicating that the patient had a very low risk for HIT. Despite differences in study design, population, setting, index test, reference standard, and measured outcomes, this study provides valuable information about HIT diagnostics and proposes CDS as a new diagnostic tool.^28^

Our results confirm that the number of patients needing antibody testing can be reduced to half if the recommended diagnostic algorithm starting with the 4Ts score is correctly used. However, 2 main problems remain. First, the sensitivity of the algorithm is limited. Therefore, a relevant proportion of patients with HIT are missed, and patients with HIT are exposed to major risks if untreated. Approximately 50% of untreated patients experience severe thromboembolism, which is associated with a high mortality rate.^1,3,4,29^ This challenges the current guidelines. Second, the current algorithm classifies a substantial number of patients as HIT positive despite being HIT negative. These patients are treated with alternative anticoagulants because functional tests are often not available or only available after a few days.^8^ However, the risk of severe bleeding is very high in these patients, exceeding 40%.^30^

The question is how to deal with the problem in daily practice? As an example of a CDS, we validated an easy-to-use machine-learning algorithm for patients with suspected HIT using the same cohort and implemented it online.^18,31^ Diagnostic machine-learning algorithms can integrate and model various clinical and laboratory information while accounting for complex interactions. The TORADI-HIT algorithm was substantially more accurate than the currently recommended diagnostic algorithm. The algorithm reduces the number of patients with false-positive and false-negative results, so that functional assays are necessary in approximately 10% of HIT patients only. Prospective cohort studies are currently running to validate the algorithm in other settings and situations.

### Strengths and Limitations

This study has strengths and limitations. The most important strength of our study is that it reflects clinical practice. Consecutive patients were included rather than selected samples, thus including the full spectrum of disease, including patients with mild disease and disorders mimicking HIT. The study was conducted following a detailed protocol defining all collection processes, ensuring complete and accurate data. An established and internally validated washed platelet functional test was conducted in all patients as the reference standard.^17^ Furthermore, we included a large number of patients, thus ensuring the appropriate power for analysis.

An important limitation of our study is, however, that most patients were included in tertiary hospitals in Switzerland, and we cannot fully exclude that the results would be different in other settings and health care institutions. We may have missed a small proportion of patients whose 4Ts score was solely determined by the treating physician without consulting the laboratory or the consultant team. In our experience, however, this is rarely the case, at most in very low-risk patients, and this would not have led to an improvement in test performance and thus would not have changed the overall conclusion of the study. Another limitation is that the scoring of the 4Ts score (joint scoring attending physician and consultancy service) might differ in other settings and that some scores were corrected in case of gross errors. However, this would further limit the performance of the tests examined, thus supporting the overall conclusion. In addition, with the present study design we were only able to examine the initial part of the algorithm and not the complete algorithm including the functional test. As a further comment, we have only included the initial determination of the 4Ts score in the calculation. It is possible that a redetermination the following day could have corrected some false negatives. However, since we were interested in the initial performance and the associated clinical decisions, we did not do consider these scorings for the purpose of this study. Of note, a smaller subset of the reported results was required in other manuscripts that answered different research questions.^18,19^

## Conclusions

This study found that in clinical practice only about half of all cases clinically suspected of HIT require antibody testing using the currently recommended diagnostic algorithm. However, a relevant number of patients were not correctly classified, potentially leading to delayed diagnosis as well as a delay in change of the anticoagulant. This exposes these often-vulnerable patients to the risk of serious thromboembolic complications or bleeding complications due to overtreatment with risky anticoagulants. The utilization of a CDS can potentially improve the diagnostic algorithms for HIT.

## Notes

Supplement 1. **eTable.** Baseline Characteristics of Patients With Low 4T Score (0-3), But Positive Heparin-Induced Activation Platelet Test **eFigure.** Participant Flow

Supplement 2.
Data Sharing Statement
