# Supplementary material for: Accuracy of Diagnosing Heparin-Induced Thrombocytopenia
Source: JAMA Netw Open. 2024 Mar 26;7(3):e243786. doi: 10.1001/jamanetworkopen.2024.3786 (PMC10966416; doi:10.1001/jamanetworkopen.2024.3786)
Supplement: Supplement 1. — eTable. Baseline Characteristics of Patients With Low 4T Score (0-3), But Positive Heparin-Induced Activation Platelet Test eFigure. Participant Flow [file jamanetwopen-e243786-s001.pdf]

Supplemental Online Content

Larsen EL, Nilius H, Studt J, et al. Accuracy of diagnosing heparin-induced thrombocytopenia. *JAMA Netw Open.* 2024;7(3):e243786. doi:10.1001/jamanetworkopen.2024.3786

eTable. Baseline Characteristics of Patients With Low 4T Score (0-3), But Positive Heparin-Induced Activation Platelet Test

eFigure. Participant Flow

This supplemental material has been provided by the authors to give readers additional information about their work.

© 2024 Larsen EL et al. *JAMA Network Open.*

eTable. Baseline Characteristics of Patients With Low 4T Score (0-3), But Positive Heparin-Induced Activation Platelet Test

	**Subjects** (n=10)
Female sex (n=)	1
Male sex (n=)	9
Age (years)	60 (55 to 67)
Setting (n=)	
Intensive care unit	2
Internal medicine	1
Major trauma	0
Postoperative - cardiovascular surgery	6
Postoperative - other surgery	1
Other	0
4Ts score (n=)	
3	7
2	3
0-1	0
Thrombosis (n=)	
Deep vein thrombosis	0
Pulmonary embolism	0
Other venous thrombosis	1
Stroke	0
Myocardial infarct	0
Skin necrosis	0
Other arterial thrombosis	1
No	8
Heparin administration last 2 weeks (n=)	
Unfractionated heparin	9
Low-molecular weight heparin	7

Age is presented as median with interquartile range.

© 2024 Larsen EL et al. *JAMA Network Open.*

eFigure. Participant Flow

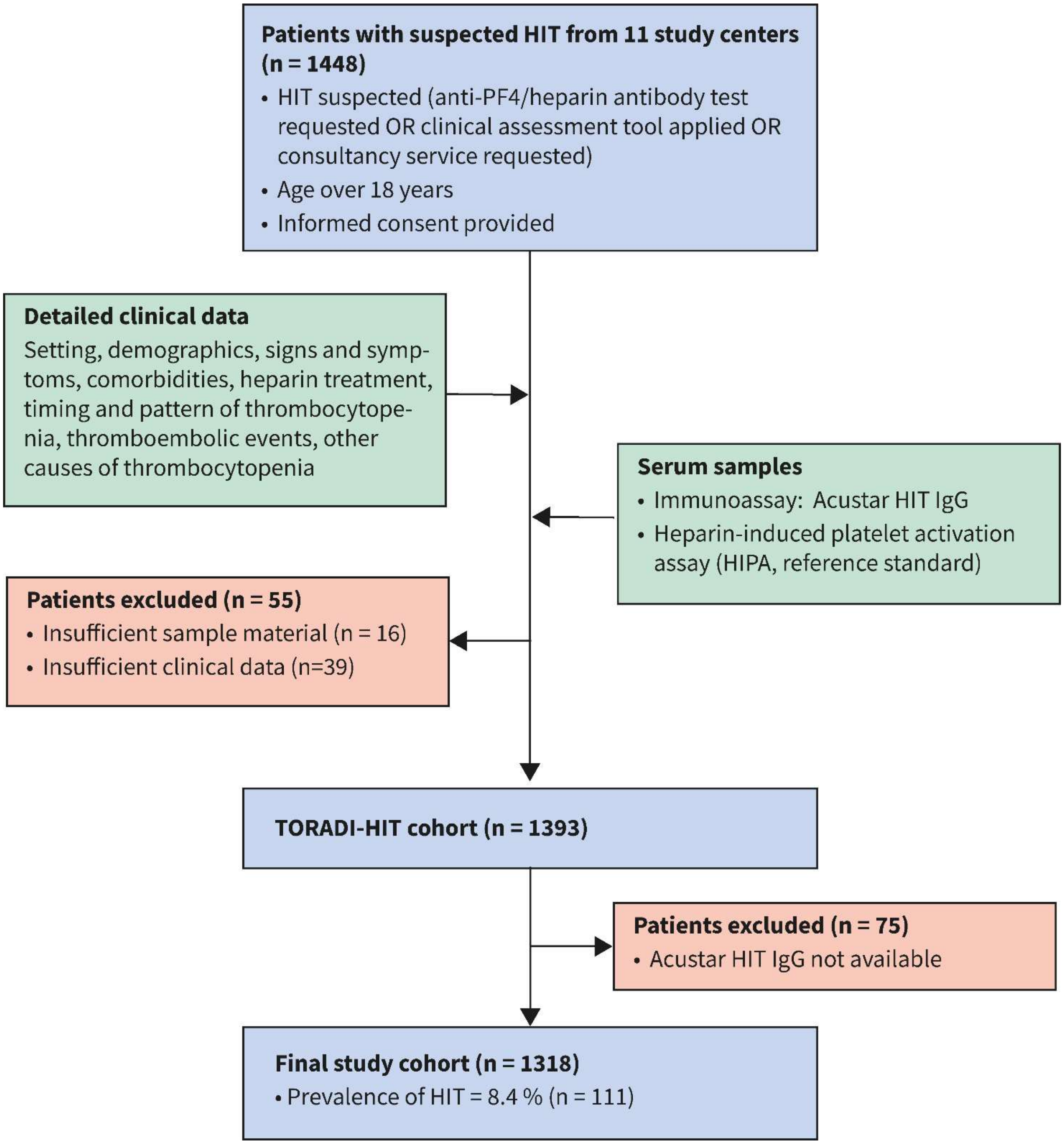

Abbreviation: HIT, heparin-induced thrombocytopenia.

© 2024 Larsen EL et al. *JAMA Network Open.*
