# Supplementary material for: Accuracy of Diagnosing Heparin-Induced Thrombocytopenia
Source: JAMA Netw Open. 2024 Mar 26;7(3):e243786. doi: 10.1001/jamanetworkopen.2024.3786 (PMC10966416; doi:10.1001/jamanetworkopen.2024.3786)
Supplement: Supplement 2. — Data Sharing Statement [file jamanetwopen-e243786-s002.pdf]

Data Sharing Statement

Larsen. Accuracy of Diagnosing Heparin-Induced Thrombocytopenia. *JAMA Netw Open*. Published March 26, 2024. doi:10.1001/jamanetworkopen.2024.3786

Data
Data available: Yes
Data types: Deidentified participant data
How to access data: michael.nagler@insel.ch
When available: With publication

Supporting Documents
Document types: None

Additional Information
Who can access the data: Researchers whose proposed use of the data has been approved.
Types of analyses: All analyses.
Mechanisms of data availability: With investigator approval, after approval, with signed data access agreement.
Any additional restrictions: N/A
